# Effects of Dietary Ramie Powder at Various Levels on the Production Performance, Serum Biochemical Indices, Antioxidative Capacity, and Intestinal Development of Laying Hens

**DOI:** 10.3389/fphys.2021.823734

**Published:** 2022-02-15

**Authors:** Xin Wang, Yang Liu, Hao-Han Zhao, Yong-Mei Wu, Chun-Jie Liu, Guang-Ying Duan, Yan-Zhou Wang, Tou-Ming Liu, Peng Huang, Ying-Hui Li, Zhi-Yong Fan, Hua-Jiao Qiu, Si-Yuan Zhu, Qian Lin

**Affiliations:** ^1^Institute of Bast Fiber Crops, Chinese Academy of Agricultural Sciences, Changsha, China; ^2^College of Animal Science and Technology, Hunan Agricultural University, Changsha, China; ^3^Hunan Deren Husbandry Technology Co., Ltd., Changde, China

**Keywords:** ramie, laying production performance, serum biochemical indices, antioxidative capacity, intestinal development

## Abstract

The purpose of this study was to investigate the effects of ramie (0, 3, 6, and 9%) included in diets on production performance, antioxidative capacity, serum biochemical indices, and intestinal development of laying hens. A total of 432 Lohmann commercial laying hens were randomly allotted to one of four dietary treatments and fed for 6 weeks. The results showed that the inclusion of ramie had no negative effects on laying performance, and increased (quadratic, *P* < 0.05) the laying rate with the highest value in the 6% ramie group. However, ramie content in the diet up to 9% reduced the apparent metabolic energy, dry matter, and organic matter apparent digestibility of laying hens compared with those in the 3% ramie group. The content of high-density lipoprotein (HDL-C) in serum was increased (*P* < 0.05), but the activity of aspartate aminotransferase (AST) was decreased (*P* < 0.05) by dietary ramie supplementation. As the dietary ramie level increased, the activity of serum glutathione peroxidase (GSH-Px) was increased quadratically (*P* < 0.05). Compared with control, 3% ramie group significantly increased (*P* < 0.01) liver total superoxide dismutase (SOD) activity. Meanwhile, the addition of 3∼6% ramie powder increased (*P* < 0.05) villus height of jejunum and villus height/crypt depth (V/C) of ileum, which reflected the intestinal promotional effect of ramie powder. In conclusion, ramie in a diet of less than 9% might protect the liver and improve the antioxidative capacity with no detrimental impacts on the laying hens. Moreover, it could promote the intestinal mucosal structure and have a positive impact on the intestine health of the laying hens.

## Introduction

Soybean meal was the main protein source of feed in China, 70% of which were imported from abroad (Yin et al., 2019). The shortage of traditional feed resources and rising prices were important factors that restricted the development of animal husbandry in recent years. Accordingly, new high-quality feed ingredients were in great need to reduce the dependency on imported protein feed, lower the feed cost, and keep or even improve the qualities of livestock and poultry products.

Ramie (*Boehmeria nivea*), also known as “Chinese grass,” is a perennial herb of the ramie family in the Urticaceae family Ramie family. With the features of fast moisture absorption and good air permeability, ramie was used as a raw material for the production of textiles (Ni et al., 2018). However, due to the low utilization rate, a large number of ramie by-products were discarded, resulting in a waste of resources (Liu et al., 2013). The nutritional values of ramie tender stems and leaves were similar to that of alfalfa, with high protein content (about 20.00% of dry matter, DM) and moderate neutral detergent fiber (NDF), reasonable amino acid composition (especially lysine, slightly more than 1.00% of DM) (Lee et al., 2009). In addition, the tender stems and leaves of ramie were edible and could be used as medication (Wang et al., 2019). Ramie belongs to the genus Ramie in the Urticaceae family and was a potential vegetable protein feed. Studies showed that feeding mice with nettle plants, which also belonged to the Urticaceae family, could significantly reduce the lipid metabolism and reduce plasma total cholesterol and triglyceride content (Avcı et al., 2006). Using ramie leaf extract could improve the blood lipid status of db/db obese mice and reduce the weight of adipose tissue (Lee et al., 2014). Moreover, previous works of literature indicated that ramie leaves had several flavonoids and polyphenols which possessed antioxidative effects *in vitro* (Chen et al., 2014). However, to our knowledge, no relevant research was conducted to test the influence of ramie treatment on hens. Therefore, the objective of this study was to evaluate the effects of varying levels of dietary ramie powder on the production performance, serum biochemical indicators, antioxidant capacity, and intestinal development of laying hens.

## Materials and Methods

### Ramie Powder Preparation

The ramie powder was prepared as described by Li et al. (2018). Briefly speaking, the leaves and tender tops were cut and collected when the ramie plant (*Boehmeria nivea* cv. Qingsizhu No. 1) grew to about 60 cm, and dried immediately at 60°C for 4 days in a forced-air oven until the water content dropped to 7%. Then the dried ramie materials were pulverized by a milling machine (SRL-Z 500, Zhangjiagang Sevenstars Machinery Co., Ltd., Zhangjiagang, China) to powders with particle size less than 1.5 mm. The processed ramie powders were then packed in seized bags and kept in a light-resistant place for further use.

### Animals and Experimental Details

All the experimental procedures were approved by the Animal Care Committee of the Institute of Bast Fiber Crops, Chinese Academy of Agricultural Sciences, Changsha, China. A total of 432 34-week-old Lohmann Commercial laying hens were randomly divided into four groups, with six replicates of 18 birds each. The diets for the four groups were control diet (corn-soybean meal diets, SBM), 3, 6, and 9% ramie powder substituted SBM, respectively. All the diets were formulated to contain similar levels of CP and to meet recommendations of the National Research Council (NRC) for laying hens (1994) as shown in Table 1. The hens were raised in ladder cages with one bird in each cage. After a 2-week adaption, the main experiment started and lasted for 6 weeks. The egg production, body weight, and feed intake of each laying hen were measured on the first day of the experiment, and no statistical differences in the production performance were found among treatments. Free water and feed were provided for all hens. The average temperature was 25 ± 2°C in the house of laying hens during the experimental period. The light time was according to the standard light procedure of commercial laying hens, which was 16 h of light per day, until the end of the experiment.

**TABLE 1:** Diet formulation and calculated nutrients (as fed basis).

Items	Control	Ramie power supplementation concentration in diets		
		3%	6%	9%
**Ingredients, %**				
Corn	51.61	51.34	51.23	51.03
Soybean meal	30.78	29.76	28.66	27.59
Rice husk	3.31	2.21	1.09	0.00
Ramie powder	0.00	3.00	6.00	9.00
Oil	2.85	2.50	2.10	1.73
Limestone	8.45	8.19	7.92	7.65
Premix	3.00	3.00	3.00	3.00
Total	100	100	100	100
**Nutrient levels (%)**				
ME (Mcal/kg)	2.75	2.75	2.75	2.75
Crude protein (%)	16.98	17.00	17.00	17.01
Crude fiber	4.44	4.44	4.44	4.45
Calcium (%)	3.50	3.50	3.50	3.50
Total phosphorus (%)	0.53	0.53	0.53	0.53
Lysine (%)	0.95	0.95	0.94	0.93
Methionine (%)	0.36	0.35	0.35	0.34

*^a^The premix provided the following (per kilogram of complete diet): vitamin A 6,000 IU, vitamin D3 2,500 IU, vitamin E 25 mg, vitamin K3 2.25 mg, vitamin B1 1.8 mg, vitamin B2 7 mg, vitamin B6 4 mg, vitamin B12 0.2 mg, D-pantothenic acid 12 mg, nicotinic acid 35 mg, biotin 0.14 mg, folic acid 0.8 mg, Cu (as copper sulfate) 11 mg, Zn (as zinc sulfate) 70 mg, Fe (as ferrous sulfate) 60 mg, Mn (as manganese sulfate) 115 mg, Se (as sodium selenite) 0.30 mg, and I (as potassium iodide) 0.4 mg.*

*^b^Nutrient levels are calculated values.*

At the end of the experiment, blood samples were taken from hens *via* the wing vein of hens (two birds/replicate, 12/treatment). One blood sample was collected from the left-wing vein of each hen in vacuum blood collection tubes. The whole blood was coagulated in a tube at room temperature and centrifugated at 3,500 rpm for 15 min. The serum samples were separated and stored at −20°C until it was used for the measurement of antioxidative and biochemical indices.

After blood collection, the hens were euthanized by carbon inhalation. After cervical dislocation, duodenum, jejunum, and ileum tissues were quickly separated from the body in a sterile environment, and about 2 cm of the middle parts of each section were taken, cleaned gently with normal saline, and fixed in 4% paraformaldehyde solution. The liver tissue samples were taken 2 g, kept in the centrifuge tube, and frozen at −20°C for later analysis. The weights of the heart, liver, spleen, small intestine, gizzard, and proventriculus were measured and the corresponding organ indexes were calculated.

### Production Performance

During the experiment, eggs of each hen were counted and weighted at 4:30 pm daily. The feed intake for each hen was recorded weekly. Finally, the egg-laying rate was calculated as the number of total eggs produced by each hen divided by experimental days. The feed conversion ratio (FCR) was calculated as grams of total feed intake per hen divided by grams of total egg mass per hen. The egg mass was calculated as the mean egg weight times the egg-laying rate.

### Apparent Nutrient Digestibility

An extra of five laying hens were used for the analysis of apparent nutrient digestibility for experimental diets. The excreta samples were collected from each hen twice a day (8:00 am and 5:00 pm) for three consecutive days and added dilute hydrochloric acid, pooled together. Then air-dried samples were prepared for subsequent experimental analysis. At the same time, according to the regulations and requirements of Feed Sampling (China, GB/T 14699.1-2005, General Administration for Quality Supervision Inspection and Quarantine of China, 2005), the experimental feed samples were collected, about 200 g in each group were collected and packed in the sample bag, and saved to be tested. The apparent metabolic rates were determined by the endogenous indicator method (acid insoluble ash, AIA). Determination of the Hydrochloric acid-insoluble ash concentration in feed and excreta followed the method described by the regulations and requirements of Determination of Hydrochloric acid-insoluble ash in feed (China, GB/T 23742-2009, General Administration for Quality Supervision Inspection and Quarantine of China, 2009). The total tract Dry matter (DM), crude protein (CP) (N × 6.25), ether extract (EE), crude fiber (CF), organic matter (OM), ash (Ash), and calcium (Ca). Phosphorus (P) digestibility and AMEn were calculated using the following Equation 1 (Yu et al., 2021). DM, CP, EE, CF, OM, Ash, Ca, and P contents in feed and excreta were determined using methods developed by the AOAC (Association of Official Analytical Chemists). Gross energy values in feed and excreta were determined using the bomb calorimeter (IKA C1 Compact Bomb Calorimeter, IKA-Werke, Staufen, Germany).

Apparent nutrient digestibility was calculated using the following equation:


$$\mathrm{Apparent}\mathrm{nutrient}\mathrm{digestibility}=\left[1-\frac{\left(\mathrm{excreta}\mathrm{nutrient}\left(\text{g}/\text{kg}\right)\right)/\left(\mathrm{excreta}\mathrm{AIA}\left(\text{g}/\text{kg}\right)\right)}{\left(\mathrm{feed}\mathrm{nutrient}\left(\text{g}/\text{kg}\right)\right)/\left(\mathrm{feed}\mathrm{AIA}\left(\text{g}/\text{kg}\right)\right)}\right]\times 100\%$$

### Serum Biochemical Indices

Serum biochemical indices, namely, alanine aminotransferase (ALT), aspartate aminotransferase (AST), and total cholesterol (T-CHO), and high-density lipoprotein (HDL-C), low-density lipoprotein (LDL-C), triglyceride (TG), glucose (GLU), total protein (TP), albumin (ALB), globulin (GLB), and uric acid (UA) were measured using assay kits (BS-200, Shenzhen Mairui Medical International Co., Ltd., China).

### Antioxidant Indices Determination

Liver tissues were retrieved from a frozen environment and separately homogenized in PBS *via* a homogenizer (LabGen 850, Cole-Parmer China, Shanghai, China). After centrifuging at 3,000 r/min for 15 min, the supernatants were collected for the examination of liver antioxidant indices levels. Serum and liver antioxidant indices, namely, total antioxidative capacity (T-AOC), total superoxide dismutase (T-SOD), glutathione peroxidase (GSH-Px), catalase (CAT), and malondialdehyde (MAD) were examined using assay kits (H249, Nanjing Jiancheng Bioengineering Institute, Suzhou, China) with an automated fluorescence instrument (MultiskanSkyHigh, Thermo Fisher Scientific, Waltham, MA, United States) following the instructions of the manufacturers.

### Determination of Intestinal Mucosa Morphological Structure

The intestinal samples were cleaned and stained with hematoxylin and eosin (HE) and finally made into paraffin sections using a microtome (RM-2235, Leica Microsystems AG Corporation Ltd., Black Forest, Germany). Then, a microscope (Van-Ox S, Olympus Corporation Co., Ltd., Washington, DC, United States) was used to randomly select multiple discontinuous fields of view to observe the slices at 40, 100, and 200 magnification times, and select typical fields of view to take pictures. The intestinal villi heights and crypt depths of each intestinal segment were analyzed and determined by Motic Images Advanced 3.2 software, then calculated the villus height/crypt depth (V/C) value at the same time.

### Statistical Analysis

The data were tested by ANOVA for the control group and experiment groups with Statistical Packages for Social Science 18.0 (SPSS 18.0) software. Orthogonal polynomial contrasts were used to determine linear and quadratic responses of levels of indices associated with production performance, serum biochemical, antioxidative capacity, and intestinal morphology to different level ramie powder. The results were expressed as arithmetic means and SEM. Statistical significance was assigned at *P <* 0.05. The *P* values between 0.05 and 0.10 were considered as a trend.

## Results

### Production Performance

The effect of dietary supplementation of ramie powder on the performance of laying hens is presented in Table 2. No mortality was found during the 6-week experimental period. The dietary ramie powder supplementation had no effects on FCR, egg weight, feed intake, or egg mass (*P* > 0.05). But it showed a trend of increasing (quadratic, *P* = 0.09) the laying rate with the highest in the 6% ramie group.

**TABLE 2:** Effects of dietary ramie supplementation on laying hen production performance.

Items	Control	Ramie supplementation concentration in diets			SEM	*P* value		
		3%	6%	9%		ANOVA	Linear	Quadratic
Egg laying rate (%)	89.05	94.16	94.82	89.34	1.053	0.090	0.651	0.015
FCR	2.20	2.12	2.13	2.21	0.038	0.852	0.990	0.386
Mean egg weight (g)	56.51	56.59	56.91	56.91	0.139	0.640	0.225	0.913
Daily feed intake (g)	114.20	109.86	111.77	113.91	1.596	0.761	0.918	0.315
Egg mass per day (g)	51.97	51.94	52.41	51.48	0.585	0.979	0.828	0.803

*^a,b^Means in the same row with different superscript letters indicate differences (p < 0.05).*

*FCR, food conversion ratio.*

### Apparent Nutrient Digestibility

As shown in Table 3, compared with 3 and 6% ramie groups, 9% ramie group significantly decreased AMEn (quadratic, *P* = 0.035), and extremely significantly decreased OM apparent digestibility (linear and quadratic, *P* = 0.008). Compared with 3% ramie group, 9% ramie group significantly decreased DM apparent digestibility (quadratic, *P* = 0.037).

**TABLE 3:** Effects of dietary ramie power supplementation on nutrient apparent metabolic rate of laying hens.

Items	Control	Ramie power supplementation concentration in diets			SEM	*P* value		
		3%	6%	9%		ANOVA	Linear	Quadratic
AMEn (MJ/kg)	12.04*^ab^*	12.77*^a^*	12.65*^a^*	11.86*^b^*	0.143	0.035	0.310	0.007
DM (%)	69.80*^ab^*	72.02*^a^*	70.64*^ab^*	67.15*^b^*	0.649	0.037	0.062	0.021
Ash (%)	48.51	52.07	52.90	49.64	1.749	0.796	0.584	0.419
OM (%)	73.01*^ab^*	75.79*^a^*	74.54*^a^*	70.08*^b^*	0.685	0.008	0.038	0.004
EE (%)	59.48	66.47	66.22	61.18	2.248	0.610	0.610	0.229
CF (%)	31.59	35.41	36.19	31.77	1.574	0.672	0.998	0.236
CP (%)	44.34	52.07	50.76	49.53	1.294	0.135	0.196	0.065
Ca (%)	65.55	66.79	69.22	65.61	0.703	0.332	0.617	0.139
P (%)	31.62	42.18	40.68	35.54	1.795	0.219	0.987	0.051

*^a,b^Means in the same row with different superscript letters indicate differences (p < 0.05).*

*AMEn, nitrogen corrected apparent metabolizable energy; DM, dry matter; Ash, ash content; OM, organic compound; EE, ether extract; CF, crude fiber; CP, crude protein.*

### Organ Indices

As shown in Table 4, none of the organ indices (cardiac index, liver index, spleen index, small intestine index, gizzard index, and proventriculus index) were affected by the dietary ramie powder supplementation on laying hens (*P* > 0.05).

**TABLE 4:** Effects of dietary ramie power supplementation on organ indices of laying hens.

Items	Control	Ramie power supplementation concentration in diets			SEM	*P* value		
		3%	6%	9%		ANOVA	Linear	Quadratic
Heart index (g/kg)	0.40	0.43	0.40	0.40	0.013	0.777	0.818	0.455
Liver index (g/kg)	2.21	2.25	2.39	2.18	0.069	0.736	0.939	0.389
Spleen index (g/kg)	0.09	0.13	0.13	0.12	0.007	0.124	0.067	0.138
Small intestine index (g/kg)	4.12	4.38	4.22	4.17	0.106	0.925	0.964	0.571
Gizzard index (g/kg)	1.90	2.17	2.10	2.23	0.070	0.382	0.146	0.628
Proventriculus index (g/kg)	0.41	0.48	0.44	0.50	0.014	0.101	0.063	0.078

*^a,b^Means in the same row with different superscript letters indicate differences (p < 0.05).*

### Serum Biochemical Parameters

The normal level of ALT, AST, T-CHO, LDL-C, HDL-C, TG, GLU, TP, ALB, GLB, and UA in laying hens ranged from 1 to 15 U/l, 65 to 270 U/l, 2 to 7 mmol/l, 0.2 to 4 mmol/l, 0.3 to 1.5 mmol/L, 7 to 28 mmol/l, 7 to 15 mmol/l, 42 to 85 g/l, 18 to 26 g/l, 23 to 58 g/l, and 177 to 440 μmol/l, respectively (Zhang et al., 2020; Lee et al., 2021; Lu et al., 2021; Tao et al., 2021; Zhu et al., 2021). As shown in Table 5, the dietary ramie powder supplementation showed a trend in decreasing (quadratic, *P* = 0.095) the concentration of ALT in serum of laying hens. Compared with the control group (Table 5), the serum AST concentration in the 3 and 6% ramie groups was significantly decreased by 22.24 and 14.14% (linear and quadratic, *P* = 0.018), respectively, and the serum HDL-C concentration in the 3% ramie group was significantly increased (linear and quadratic, *P* = 0.025).

**TABLE 5:** Effects of dietary ramie power supplementation on serum biochemical indices of laying hens.

Items	Control	Ramie power supplementation concentration in diets			SEM	*P* value		
		3%	6%	9%		ANOVA	Linear	Quadratic
ALT (U/l)	7.91	3.27	4.59	5.48	0.731	0.095	0.229	0.042
AST (U/l)	321.13*^a^*	249.72*^b^*	258.44*^b^*	275.71*^ab^*	10.413	0.018	0.030	0.014
T-CHO (mmol/l)	2.82	2.49	2.65	2.76	0.110	0.731	0.823	0.324
LDL-C (mmol/l)	3.40	2.51	3.23	3.14	0.205	0.464	0.744	0.272
HDL-C (mmol/l)	0.46*^b^*	0.73*^a^*	0.66*^ab^*	0.64*^ab^*	0.038	0.025	0.017	0.060
TG (mmol/l)	17.75	12.25	17.71	16.51	1.218	0.354	0.750	0.217
GLU (mmol/l)	10.19	11.05	11.46	10.75	3.08	0.955	0.706	0.693
TP (g/l)	74.72	76.52	81.68	74.15	6.66	0.449	0.581	0.258
ALB (g/l)	23.01	23.02	25.66	22.41	1.87	0.112	0.598	0.186
GLB (g/l)	51.71	53.51	56.03	51.75	5.72	0.714	0.638	0.371
UA (μmol/l)	85.42	70.84	59.71	84.56	22.55	0.412	0.520	0.147

*^a,b^Means in the same row with different superscript letters indicate differences (p < 0.05).*

*ALT, glutamic-pyruvic transaminase; AST, glutamic-oxaloacetic transaminase; T-CHO, total cholesterol; HDL-C, high-density lipoprotein; LDL-C, low-density lipoprotein; TG, triglyceride; GLU, glucose; TP, total protein; ALB, albumin; GLB, globulin; UA, uric acid.*

### Antioxidant Indices

The normal level of T-AOC, T-SOD, GSH-Px, CAT, and MDA in laying hens ranged from 0.6 to 5 U/ml, 50 to 450 U/ml, 180 to 500 U/ml, 3 to 12 U/ml, 0.9 to 6 U/ml, respectively (Li et al., 2019; Yu et al., 2020; Chen et al., 2021; Fu et al., 2021; Gu et al., 2021; Zhu et al., 2021). The antioxidation indices of the serum antioxidant are shown in Table 6. Compared with the control group and 9% ramie group, 3% ramie group significantly increased the serum GSH-Px activity of laying hens (quadratic, *P* = 0.026). There were no significant differences in other serum indices with different ramie meal levels (*P* > 0.05). Serum indices (T-AOC, T-SOD, and CAT) in ramie powder groups were slightly higher than those in the control group, MAD was slightly lower than the control group. Compared with other groups (Table 7), 6% ramie group significantly increased the activity of T-SOD in the liver (linear and quadratic, *P* = 0.003), but there were no significant differences in other indices (*P* > 0.05).

**TABLE 6:** Effects of dietary ramie power supplementation on serum antioxidant indices of laying hens.

Items	Control	Ramie power supplementation concentration in diets			SEM	*P* value		
		3%	6%	9%		ANOVA	Linear	Quadratic
T-AOC (U/ml)	0.35	0.38	0.39	0.37	0.020	0.921	0.728	0.585
T-SOD (U/ml)	29.69	49.54	49.36	34.81	6.128	0.682	0.923	0.266
GSH-Px (U/ml)	294.36*^b^*	531.43*^a^*	405.44*^ab^*	362.26*^b^*	35.763	0.026	0.924	0.011
CAT (U/ml)	10.43	16.64	11.78	11.77	1.258	0.374	0.939	0.254
MDA (nmol/ml)	2.03	0.97	1.56	1.20	0.177	0.183	0.199	0.306

*^a,b^Means in the same row with different superscript letters indicate differences (p < 0.05).*

*T-AOC, total antioxidative capacity; T-SOD, total superoxide dismutase; GSH-Px, glutathione peroxidase; CAT, catalase; MAD, malondialdehyde.*

**TABLE 7:** Effects of dietary ramie power supplementation on liver antioxidant of laying hens.

Items	Control	Ramie power supplementation concentration in diets			SEM	*P* value		
		3%	6%	9%		ANOVA	Linear	Quadratic
T-AOC (U/g)	0.51	0.60	0.64	0.55	0.035	0.729	0.830	0.299
T-SOD (U/g)	161.17*^b^*	204.67*^b^*	296.66*^a^*	201.39*^b^*	17.245	0.003	0.024	0.002
GSH-Px (U/g)	12.61	14.50	15.54	12.60	0.601	0.182	0.716	0.051
CAT (U/g)	31.62	32.54	34.04	32.50	0.944	0.916	0.784	0.601
MDA (nmol/g)	31.56	31.55	23.25	30.81	3.053	0.830	0.827	0.605

*^a,b^Means in the same row with different superscript letters indicate differences (p < 0.05). T-AOC, total antioxidative capacity; T-SOD, total superoxide dismutase; GSH-Px, glutathione peroxidase; CAT, catalase; MAD, malondialdehyde.*

### Intestinal Mucosa Morphological Structure

The morphological structure of the intestinal mucosa of laying hens is shown in Table 8. Ramie powder supplement of 3 or 6% increased the villus height of jejunum by 27.76 or 27.36% (linear and quadratic, *P* = 0.048), and V/C value of ileum (linear and quadratic, *P* = 0.018), compared with the control. There was a tendency to increase the value of V/C (quadratic, *P* = 0.096) in the jejunum in different ramie powder levels.

**TABLE 8:** Effects of dietary ramie power supplementation on morphological structure of intestinal mucosa of laying hens.

Items		Control	Ramie power supplementation concentration in diets			SEM	*P* value		
			3%	6%	9%		ANOVA	Linear	Quadratic
Duodenum	Villus height (μm)	581.79	635.02	595.56	612.77	22.504	0.900	0.771	0.739
	Crypt depth (μm)	75.44	75.71	75.58	78.82	2.710	0.976	0.730	0.819
	V/C	7.52	8.31	7.68	7.71	0.300	0.814	0.983	0.517
Jejunum	Villus height (μm)	416.59*^b^*	532.25*^a^*	530.56*^a^*	496.66*^ab^*	20.653	0.048	0.037	0.038
	Crypt depth (μm)	72.75	65.27	67.06	72.63	2.027	0.520	0.867	0.169
	V/C	5.42	8.38	7.49	6.84	0.467	0.096	0.277	0.040
Ileum	Villus height (μm)	274.78	309.33	452.70	336.33	29.520	0.122	0.144	0.135
	Crypt depth (μm)	53.75	44.91	57.40	50.41	2.086	0.213	0.983	0.576
	V/C	5.06*^b^*	6.68*^a^*	7.62*^a^*	6.48*^ab^*	0.332	0.018	0.033	0.010

*^a,b^Means in the same row with different superscript letters indicate differences (p < 0.05).*

*V/C, villus height/crypt depth.*

## Discussion

The shortage of protein feed is one of the critical factors restricting the development of the livestock industry in China. Due to the harsh weather conditions, it is hard to grow high-quality forages (alfalfa) in south China. However, as an important economic crop, over 90% of the world’s ramie is grown in southern China (Kipriotis et al., 2015). The nutritional value of ramie is similar to that of alfalfa (Dai et al., 2019). Furthermore, ramie leaves, which are used for medicinal and edible purposes, are effective in reducing serum cholesterol, improving the meat quality of farmed animals (Avcı et al., 2006; Tang et al., 2021).

Safety was always the top priority to consider when utilizing untraditional resources for animal feed purposes. According to the data of this study, it was the first to report that ramie had no adverse effect on the overall production performance of hens. Many pieces of research showed that egg production has been increased in response to nettle supplementation, such as *Urtica dioica* and *Urtica cannabina* (Loetscher et al., 2013; Zhang et al., 2020). The herb *Boehmeria nivea*, as a perennial dicotyledon of the Urticaceae family, may have the same effect. Similarly, it was reported that egg production remained unchanged (Wang and Jie, 2012) or even tended to increase (Luo et al., 1989) with the supplement of ramie powder. In line with these studies, the supplementation of 3 and 6% ramie powder showed a trend in increasing egg production. Ramie (*Boehmeria nivea*) is rich in cellulose, flavonoids compounds, polyphenol compounds, vitamin C, and minerals. A large number of studies showed that scientific applications of vitamins and trace elements could improve animal performance and feed return (Abd El-Hack et al., 2017; Han et al., 2017). Perhaps, ramie supplemented vitamins and trace elements in the diet, ensured the full price of diet nutrition, thereby improving laying hens production performance. On the other hand, it might be the antioxidant properties of ramie. Recent studies showed that adding flavonoids to the diet could improve the performance of laying hens by improving the body’s antioxidant capacity, reducing the occurrence of oxidative stress, and promoting the absorption of nutrients in the intestine (Brisibe et al., 2008; Galal et al., 2008; Seven, 2008; Liu et al., 2014; Zhu et al., 2021).

Ramie has a relatively high crude fiber content, which will affect the digestion and utilization of other nutrients as the dosage increase. Studies showed that the increase of dietary fiber affected the digestibility of CP, EE, and AMEn in varying degrees, and reduced production performance (Tabook et al., 2006; Liu et al., 2010). In our study, dietary substituted with 9% ramie powder significantly decreased AMEn and DM apparent digestibility of laying hens (*p <* 0.05), and extremely significantly decreased the apparent digestibility of OM (*p <* 0.01) compared with 3 and 6% ramie groups. However, the dietary substitution of 3 and 6% ramie had no significant effect on the apparent metabolic rate of nutrients. In conclusion, the feed digestion and utilization of laying hens could be promoted if the inclusion of ramie powder was between 3 and 6%, but it might have an adverse effect if the ramie powder substitution rate was too high. This was basically the same for geese (Liu et al., 2010). Although high CP was beneficial to the growth of laying hens, high CF and antinutritional factors in ramie could have negative impacts. Compared with other animals, laying hens had relatively low utilization of fiber forage. The higher CF content was in the diet, the greater the physical shielding effect of the diet was on digestive enzymes, which hindered the full contact of digestive enzymes and nutrients (fat, starch, protein, etc.) in the intestine (Guzmán et al., 2016), and greatly affected the apparent digestibilities of various nutrients in the diet (Knudsen, 2001).

High-density lipoprotein is involved in cholesterol reverse transport and is a key lipoprotein that maintains lipid balance in the cardiovascular system (Pirillo et al., 2019). As a complex of lipids and proteins, HDL-C not only participates in regulating lipid metabolism but also inhibits thrombosis and inflammation and regulates glucose metabolism (Feng et al., 2020). In normal conditions, two common serum enzymes (AST and ALT) only existed in cells because of the barrier function of cell membranes. When tissues (especially the liver) were damaged, excessive oxygen free radicals were generated and invaded the cell membranes that increasing the permeability of the cell membrane, which in turn led to the escape of AST and ALT into the serum (Ma et al., 2021). Ramie ethanol extract could significantly reduce serum TC and LDL-C levels of mice, and significantly increase HDL-C level (*p <* 0.05) (Lee et al., 2014). In this study, 3 and 6% ramie groups had significantly lower content of AST in serum compared to the control group (*p* < 0.05), indicating that ramie played a protective role. Chlorogenic acid was the main phenolic compound in ramie ethanol extract (Tan et al., 2014), and had a variety of biological effects such as antioxidant, antidiabetic, and antilipid effects (Ong et al., 2013). The serum HDL-C content in the 3% ramie group was significantly increased (*p <* 0.05), which may be due to the reason that polyphenols in ramie leaves (chlorogenic acid, etc.) inhibited glucose 6-phosphate translocase, that modulated hepatic glucose 6-phosphatase system and regulated the homeostasis of blood glucose (Arion et al., 1998).

The animal bodies produced some free radicals in the metabolism process. Among them, oxygen-free radicals could cause lipid peroxidation of polyunsaturated fatty acids in biofilms. With the increase of lipid peroxidation reaction, the capacity of antioxidant decreased, and the original balance between the antioxidant system and the production of pro-oxidants were broken, leading to oxidative stress in the body (Seifried et al., 2007; Lin et al., 2008; Poljsak et al., 2013). Oxidative stress damaged the biological macromolecules (DNA, lipids, proteins, etc.) in the body, affecting animal health and the quality of livestock and poultry products. Antioxidant defense system, included enzymatic reaction system (endogenous antioxidant enzymes, such as SOD, GSH-Px, and CAT) and non-enzymatic reaction system (water and lipid-soluble compounds, such as vitamin C and polyphenols), the latter of which were rich in ramie. Polyphenols and flavonoids in ramie leaves were considered to be the main bioactive components with antioxidant capacity (Lee et al., 2014). Moreover, ramie also promoted antioxidant enzyme activities, decreased lipid peroxidation level, and improved the body’s antioxidant capacity (Li et al., 2019). Some oxygenated lipids could regulate the production of antioxidants (Mavangira and Sordillo, 2018). Organic acids accounted for the largest proportion of volatile components in ramie leaves, and the content of polyunsaturated fatty acids was more than 45%. Oxylipid was the abbreviation of various lipid metabolites produced in the oxidative metabolism of PUFA. In this study, 3% ramie group significantly increased the content of GSH-Px in serum (*p <* 0.05), 6% ramie group extremely significantly increased the content of T-SOD in the liver (*p <* 0.01) compared with the control group. It might be because that ramie as a feed source improved the activities of antioxidant enzymes and increased the intake of PUFA and polyphenols, thereby enhancing the antioxidant capacity of laying hens, extending the laying period, and improving the production performance.

Villus height, crypt depth, and the value of villus height/crypt depth (V/C) were critical indicators to evaluate the digestion and absorption function of the intestine. Intestinal villi were the main tissues that absorb nutrients. As the height of the villi increased, the ability of the body to absorb nutrients was also enhanced. The depth of the crypt could reflect the renewal speed of intestinal epithelial cells. The shallower the depth, the slower the differentiated intestinal epithelial cells migrated to the upper part of the villi. It indicated that the higher the maturation rate of intestinal epithelial cells were, the stronger the absorption function they had. The value of V/C comprehensively reflected the functional status of the intestine. The increase in V/C value indicated that the intestinal lining area was larger, the structure of the intestinal mucosa was improved, and the digestion and absorption functions were enhanced (Yason et al., 1987; Lin et al., 2017). Medicinal plants rich in polyphenols could effectively inhibit the growth of harmful bacteria in the gut and improve the composition of the gastrointestinal microbiota. Silage ramie significantly increased ileum villus height and V/C value of geese (*p <* 0.05) (Hou et al., 2018), which was consistent with our study that 3 or 6% ramie powder substitution significantly increased the villus height of jejunum and the V/C value of ileum. These results indicated that ramie could improve the intestinal mucosal morphology of laying hens. It might be because ramie, a medicinal plant rich in polyphenols, improved the antioxidant capacity of the whole gastrointestinal tract (Bonetti et al., 2016). On the other hand, ramie might inhibit the growth of harmful bacteria in the intestinal tract and improve the composition of gastrointestinal microbiota (Lee et al., 2001).

## Conclusion

In summary, ramie would not cause adverse effects on the organs of Lohmann commercial laying hens. The substitution rate between 3 and 6% of ramie was beneficial to reduce blood lipids, improve antioxidant capacity and intestinal mucosa structure, and promote egg-laying rate. However, with the substitution rate of ramie exceeding 9%, it could affect the apparent metabolizable energy and the nutrient utilization of dry matter and organic matter of the laying hens. Considering all indicators, the optimum substitution rate of dietary ramie for laying hens was 6%.

## Data Availability Statement

The original contributions presented in the study are included in the article/supplementary material, further inquiries can be directed to the corresponding authors.

## Ethics Statement

The animal study was reviewed and approved by all the experiment procedures were approved by the Animal Care Committee of the Institute of Bast Fiber Crops, Chinese Academy of Agricultural Sciences, Changsha, China.

## Author Contributions

QL, S-YZ, H-JQ, and Z-YF designed and conducted the study. XW, H-HZ, G-YD, and Y-MW conducted the animal experiment. C-JL, Y-ZW, T-ML, PH, and Y-HL conducted the detection and analysis works. XW, YL, and QL prepared the manuscript draft. All authors participated in the discussion and editing of the manuscript.

## Conflict of Interest

YL, Y-MW, G-YD, and QL was employed by Hunan Deren Husbandry Technology Co., Limited. The remaining authors declare that the research was conducted in the absence of any commercial or financial relationships that could be construed as a potential conflict of interest.

## Publisher’s Note

All claims expressed in this article are solely those of the authors and do not necessarily represent those of their affiliated organizations, or those of the publisher, the editors and the reviewers. Any product that may be evaluated in this article, or claim that may be made by its manufacturer, is not guaranteed or endorsed by the publisher.
